# The presence of *Mycoplasma bovis* in colostrum

**DOI:** 10.1186/s13567-020-00778-w

**Published:** 2020-04-16

**Authors:** Linde Gille, Julien Evrard, Jozefien Callens, Karlien Supré, Fabien Grégoire, Filip Boyen, Freddy Haesebrouck, Piet Deprez, Bart Pardon

**Affiliations:** 1 Department of Large Animal Internal Medicine, Faculty of Veterinary Medicine, Ghent University, Merelbeke, Belgium; 2 Regional Association for Animal Identification and Health (ARSIA), Ciney, Belgium; 3 Animal Health Service Flanders (DGZ-Vlaanderen), Torhout, Belgium; 4 Flanders Milk Control Centre (MCC-Vlaanderen), Lier, Belgium; 5 Department of Pathology, Bacteriology and Avian Diseases, Faculty of Veterinary Medicine, Ghent University, Merelbeke, Belgium; 6 Present Address: Clinical Department of Production Animals, Faculty of Veterinary Medicine, University of Liege, Liege, Belgium; 7 Present Address: Zoetis SA, Zaventem, Belgium

## Abstract

In herds with *Mycoplasma bovis* circulation, colostrum is often considered infectious. However, in contrast to milk, the presence of *M. bovis* in colostrum was not previously evidenced. In this survey, the presence of *M. bovis* DNA was determined with real-time PCR in 368 colostrum samples from 17 herds, recently infected with *M. bovis*. Only 1.9% of the samples tested positive, with 13 herds having no positive samples and an overall within-herd prevalence of 3.2% (SD: 4.9%; Range: 0–30.0%). These results show that in infected herds *M. bovis* DNA can be retrieved in colostrum. To what extend colostrum is infectious remains to be determined.

## Introduction, methods and results

*Mycoplasma bovis* strongly contributes to economically important diseases like mastitis and pneumonia and heavily affects animal welfare and antimicrobial use in modern dairy farming [1]. Prevalence seems to be rising and increasing antimicrobial resistance has been reported as well [2, 3].

Between animals, the major ways of *M. bovis* transmission are direct nose to nose contact and aerosol spread [1] and consumption or contact with infected milk [4]. Introduction into a herd generally happens through purchase of replacement animals [5]. However, several other, currently under documented, ways of *M. bovis* introduction might exist. Troubling recent illustrations are the introduction of *M. bovis* in two Finnish herds by use of contaminated artificial insemination semen [6], and the first detection of *M. bovis* in New Zealand in July 2017 [7, 8]. In the latter outbreak, import of embryos, feed, fomites, semen and other animal species were investigated as sources of this introduction, since no live cattle were imported since 2013, but to date the source remains unidentified [8]. In the current mindset of reducing antimicrobial use and improving animal health, it is imperative to prevent *M. bovis* introduction in farms and countries alike.

Colostrum was mentioned as a possible source of *M. bovis* in the past [9, 10], but to the author’s knowledge, no studies on the prevalence of *M. bovis* in colostrum are currently available. Despite this lack of information, empirically designed *M. bovis* herd control programs often advocate the removal or (heat-) treatment of the herd’s own colostrum as a precaution measure [4]. Withholding colostrum from neonatal calves is not an option, as they depend on colostrum to bridge the period from birth until their own immunity is fully functional [11]. Purchase of colostrum from other herds holds a risk for infectious diseases, especially paratuberculosis (*Mycobacterium avium* subsp*. paratuberculosis*) [12] and will not provide the calf with herd-specific maternal immunity. Decontaminated colostrum (pasteurized or gamma irradiated) can be purchased, but this will result in a significant financial burden. Heat treatment lacks, especially in smaller farms, economical and practical feasibility due to the small amounts to be processed. Furthermore, heat treatment destroys the cytokine and cellular fraction of the colostrum, which seems to affect the calves’ recovery after antigen exposure [13]. Knowledge on the prevalence of *M. bovis* in colostrum is essential to guide farmers in the choice of which preventive or control measures are preferentially taken. Therefore, the main objective of this study was to determine the presence of *M. bovis* DNA in colostrum samples from herds with a recent *M. bovis* infection.

A survey was conducted on seventeen farms throughout Belgium. Farms were conveniently selected by the local veterinarian and samples were collected throughout 2016 and 2017. The inclusion criteria were a recent (less than one month) *M. bovis* infection in the herd, documented by either positive culture or PCR, and the willingness of the farmer to participate. Farms could be either beef, dairy or dairy-mixed type. Four beef, five dairy and eight dairy-mixed farms participated (Table 1). Group A farms all had positive BAL or lung PCR samples in calves, without information on adults, whereas group B farms had confirmed *M. bovis* infection in both adult cattle and calves. Sample size calculations were preset on the available budget, which allowed the analysis of a total of 370 samples. Based on an average herd size of 80 lactating animals [3] and with an expected prevalence of 25% of the animals shedding, ten animals needed to be sampled in each herd to detect *M. bovis* with 95% confidence. A limit of twelve samples per herd was set on thirteen farms (Group A farms). Four farms with confirmed adult *M. bovis* cases were sampled for a longer time (six to twelve months) in an attempt to better characterize potential periods of shedding (Group B farms). Colostrum samples were collected by the farmer immediately post-partum after disinfection of the teats with gauze drenched in alcohol. A composite cow sample (pooled sample of all four quarters for each cow) was taken for each cow in a 15 mL Falcon™ tube (Fisher Scientific, NH, USA). Samples were stored on farm at −20 °C until analysis. Farmers were informed on the most ideal sampling procedure and provided with the necessary material to perform this in a repeatable fashion. Because of the regional availability of two different laboratories where samples were sent to, two different real-time PCR assays were used. In both laboratories, interpretation of PCR outcome was similar: samples were considered negative, borderline positive, or positive with a Ct value of > 40, 37–40, and < 37, respectively. As the PCR protocols were used for colostrum, a non-standard medium, both protocols were verified for this matrix by use of spiked colostrum samples (10^8^ CFU/mL). The samples of Group A farms were analyzed individually for the presence of *M. bovis* DNA by real-time PCR (VetMAX™ *M. bovis* kit, ThermoFisher Scientific, MA, USA), targeting the *uvrC* gene. Before analysis, samples were mixed with PBS, centrifuged and the supernatants discarded. A mix of proteinase K/ATL buffer (Qiagen, Hilden, Germany) was added to the pellet before cell lysis. DNA was automatically extracted by use of the MagAttract 96 cador pathogen kit (Qiagen) and KingFisher™ Flex 96 Deep-Well Magnetic Particle Processor (Thermo Fisher Scientific™), according to the manufacturer. In Group B farms, pooled samples were examined using the real-time PCR Pathoproof^®^ Complete 16-kit (Thermo Fischer Scientific™) according to the supplier’s manual. Each pool consisted of five cow composite samples of cows belonging to the same herd. Samples of the *M. bovis* positive pools were analyzed individually the next day to find positive samples.

**Table 1:** Prevalence of *Mycoplasma bovis* in freshly calved cattle from recently infected herds.

Study group	Group A													Group B			
Herd number	1	2	3	4	5	6	7	8	9	10	11	12	13	14	15	16	17
Type	B	B	D	D	M	M	M	B	M	M	M	M	M	D	D	D	B
Total *n* of cattle in herd	161	139	74	103	209	121	216	152	205	245	316	433	282	587	363	311	241
% PCR Pos (p/nt)	0 (0/6)	0 (0/11)	0 (0/8)	0 (0/3)	0 (0/12)	0 (0/5)	30 (3/10)	10 (1/10)	0 (0/10)	0 (0/11)	0 (0/4)	11.1 (1/9)	0 (0/11)	0 (0/63)	2.8 (2/71)	0 (0/74)	0 (0/50)

*n* = number; % PCR Pos = Percentage of positive samples; p/nt = absolute number of samples positive over number of animals tested; Types: B = Beef farm; D = Dairy farm, M = Mixed farm.

Data were entered in Excel 2016, and analyzed by SPSS version 25 (IBM, Armonk, New York, USA). In total, 368 colostrum samples from seventeen herds were analyzed (Table 1). *M. bovis* DNA was detected in 1.9% (7/368) of these samples, obtained from four different farms. Thirteen of the 17 sampled farms did not have any *M. bovis* positive colostrum samples. On the four farms that did have positive samples, on-farm/within herd prevalence ranged between 2.8% (2/71) and 30% (3/10). The average within herd prevalence was 3.2% (standard deviation: 4.9%; range: 0–30.0%). Of the seven positive samples, five samples yielded a borderline positive Ct value (> 37 and < 40). Only one farm (2 samples) was positive in group B. No pattern of shedding over time could be identified in group B farms, as very few samples were positive.

## Discussion

This study aimed at determining the prevalence of *M. bovis* in colostrum. The study faces important limitations as we used the current, commercially available PCRs, originally manufactured for milk on colostrum samples. Diagnostic accuracy of these tests for colostrum is undocumented. We performed a limited validation to assure that positive samples are detected by spiking colostrum samples with *M. bovis*. Also, in the B group pooling of colostrum could have had an impact on the detection limit, given the high Ct value of the few positives detected, possibly other positive samples were missed. Therefore, current prevalence estimate needs to be interpreted carefully. Because colostrum samples can only be collected at one time point (just after calving), the decision was made to have the sampling performed by the farmer. Unfortunately, not all farmers complied 100% with the protocol and did not send in the twelve samples required for each farm to achieve the desired level of precision. Several PCR positive samples had a high Ct value, indicating only a marginal amount of *M. bovis* DNA present in the sample (Table 2). High Ct values could indicate the presence of other *Mycoplasma* species [14], and very high Ct values may also indicate carryover of DNA between samples [15]. Even though all farmers were instructed to take milk samples as aseptically as possible through an on-site demonstration, it is possible the actual sampling was not done *lege artis* in every case.

**Table 2:** Ct-values of positive samples after real-time PCR and their interpretation.

Source herd	Herd 7			Herd 8	Herd 12	Herd 15	
Ct value	37.14	38.87	38.2	38.36	29.14	34.3	39.8
Interpretation	Borderline	Borderline	Borderline	Borderline	Positive	Positive	Borderline

Taking these limitations into account, using these PCRs, *M. bovis* DNA was only sporadically detected in colostrum. It is unclear whether the concentration of bacteria present in colostrum would suffice to infect calves, especially in the case of marginally positive samples. Furthermore, the presence of live bacteria was not verified in this study and should be investigated further.

The main finding of this study was that *M. bovis* DNA could be detected in colostrum in a small number of samples. In the herds where a longitudinal follow-up was done, only two positive samples were found on a total of 258 samples, while *M. bovis* was still circulating in the herd during the entire study (based on sampling of clinical cases on nasal swabs or milk). A variation of colostral shedding was seen between the tested herds in this study, which could indicate differences in excretion of *M. bovis*. Hypothetically, this could be based on the time of introduction of *M. bovis* in the herd, where recently infected herds would have a higher level of shedding, concurrent with the rapid spread of a *M. bovis* strain through a seronegative population [16]. However, herd 17 was experiencing a large outbreak of *M. bovis* related disease in adult cattle at the time of sampling, after a primary introduction into the herd 1 month earlier, without any detectable shedding of *M. bovis* in the colostrum tested. Colostral shedding of *M. bovis* might also be linked to the disease form that *M. bovis* shows on a certain farm. One could suppose that farms suffering from *M. bovis* mastitis will have more shedding in milk. Given that case selection in group A was based on *M. bovis* positive tests in calves, this could have affected the isolation rate. However, group B farms were all selected based on *M. bovis* positive tests in adults. Overall, the within herd prevalence of *M. bovis* DNA in colostrum was very low, with the exception of one herd where 30% was tested positive. This might point to the fact that some individual herds are more affected, depending on the timing of infection of periparturient cows. Alternatively, it might be the consequence of false positive PCR results due to sample contamination or the presence of other *Mycoplasma* species [14, 15].

In conclusion, correct interpretation of the present results is crucial. *M. bovis* DNA is present in colostrum samples, albeit at low frequency. The DNA presence does not provide any information on the infectious risk of colostrum. Further work is needed to determine the risk of transmission trough colostrum and whether cows positive in colostrum have continued shedding in milk later in lactation.

Based on the findings of this study, farmers and veterinarians might be motivated to apply the precautionary principle and decontaminate colostrum or purchase colostrum (replacer). Discarding colostrum of cows suffering from *M. bovis* related pathologies is likely a good advice. We also recommend practitioners and farmers to avoid pooling of colostrum in infected farms. Pasteurization [9] and purchase of colostrum (replacers) are options, whereas freezing and subsequent thawing only reduces *M. bovis* concentration [17]. Acid treatment [18] of colostrum could be investigated as an alternative. Herd health advisors need to be aware that the investment cost for on-farm pasteurization and purchase of colostrum (replacers) might be high and that negative effects on herd immunity might be the consequence. Therefore, these measures are potentially not in economic equilibrium with the transmission risk via colostrum.

## Data Availability

All data on positive samples is available in the main manuscript. The full data set on PCR is available on request.
